# Songbird mesostriatal dopamine pathways are spatially segregated before the onset of vocal learning

**DOI:** 10.1371/journal.pone.0285652

**Published:** 2023-11-16

**Authors:** Malavika Ramarao, Caleb Jones, Jesse H. Goldberg, Andrea Roeser

**Affiliations:** Department of Neurobiology and Behavior, Cornell University, Ithaca, NY, United States of America; Texas Christian University, UNITED STATES

## Abstract

Diverse dopamine (DA) pathways send distinct reinforcement signals to different striatal regions. In adult songbirds, a DA pathway from the ventral tegmental area (VTA) to Area X, the striatal nucleus of the song system, carries singing-related performance error signals important for learning. Meanwhile, a parallel DA pathway to a medial striatal area (MST) arises from a distinct group of neighboring DA neurons that lack connectivity to song circuits and do not encode song error. To test if the structural and functional segregation of these two pathways depends on singing experience, we carried out anatomical studies early in development before the onset of song learning. We find that distinct VTA neurons project to either Area X or MST in juvenile birds before the onset of substantial vocal practice. Quantitative comparisons of early juveniles (30–35 days post hatch), late juveniles (60–65 dph), and adult (>90 dph) brains revealed an outsized expansion of Area X-projecting neurons relative to MST-projecting neurons in VTA over development. These results show that a mesostriatal DA system dedicated to social communication can exist and be spatially segregated before the onset of vocal practice and associated sensorimotor experience.

## Introduction

Midbrain dopamine (DA) neurons project to diverse striatal areas and contribute to reinforcement learning by signaling reward prediction error, the difference between actual and predicted reward [1]. In classic models, this DA reinforcement signal is posited to be globally broadcast throughout the brain so that an animal can learn arbitrary stimulus-response associations [2–4]. Closer examination of the pathway-specific DA signaling has shown that DA signals can vary across distinct striatal regions [5–8]. For example, medial, lateral, dorsal and ventral zones of the mesostriatal projection can exhibit distinct responses to movements and errors in reward prediction [9]. The DA projections to the posterior tail of the striatum and specific nuclear accumbens regions may even exhibit non-canonical responses to aversive stimuli [10, 11]. These studies raise the possibility that mesostriatal DA systems do not always broadcast a global scalar reinforcement signal but rather may provide a vector of evaluative feedback that targets specific parts of the animal’s motor system to produce optimal behaviors in complex environments [12].

The evidence that distinct anatomical channels of the mesostriatal system exist and may exhibit distinct aspects of evaluative feedback arises primarily from studies in adult animals. This raises a critical question: do distinct mesostriatal DA systems achieve their segregation by synaptic pruning early in life, possibly influenced by experience? If this is the case, then juvenile animals may exhibit less anatomical segregation than adults. Alternatively, segregation might be specified genetically and be evident in juveniles before the onset of learning.

Songbirds provide an ideal model system to examine mesostriatal pathway development. First, songbirds have a discrete neural circuit, the song system, which is dedicated to song learning [13]. Importantly, in male zebra finches, the song system contains a specific DA projection from VTA to Area X, the singing-specialized striatum [14, 15]. Female zebra finches do not sing and lack a well-formed song system. Past studies showed that in adults, neurons in VTA that project to Area X (VTA_X_ neurons) exhibit performance evaluation signals important for learning [16–20]. In addition, VTA_X_ neurons are distinct from VTA neurons that project to a more medial striatal region (MST) unassociated with vocal circuits [14, 15]. We have been perplexed by two aspects of the VTA-Area X and VTA-MST pathways in songbirds. First, even though VTA_X_ neurons and MST projecting VTA neurons (VTA_MST_ neurons) arborize in distinct striatal regions, they can be spatially intermingled, with cell bodies adjacent to one another [15]. Second, VTA_X_ neurons carry singing related auditory error signals, but neighboring ones that project to MST do not. In electrophysiological recordings, antidromically identified VTA_X_ neurons that exhibited error signals during singing were often recorded in the same electrode penetration, and at the same site, as VTA neurons that did not project to Area X and did not encode performance error [17, 18]. How do the neurons in VTA that encode auditory error ‘know’ to send their axons to Area X and not to MST? One possibility is that a single, global mesostriatal DA system exists early in development, but that the act of singing contributes to an experience-dependent process that causes separate pathways to form. Singing-related auditory signaling found in some VTA neurons promotes synapse formation with singing-related neurons in Area X, analogous to how early visual experience enables normal visual system development and, for example, ocular dominance columns [21]. Alternatively, the VTA_X_ projection could be genetically specified, for example, by cell-class specific markers that control axon guidance [22].

Zebra finches learn to sing through a gradual process of trial and error. In early development, until about 35 days post hatch (dph), young birds hear a tutor song and form an auditory memory, or an internal model, to be imitated [23]. Between 35–45 dph, juvenile male zebra finches begin to sing subsong, highly variable vocalizations akin to human vocal babbling [24]. Over the next 50 days, the song gradually becomes more stereotyped and similar to the tutor song, finally crystalizing by 90–100 dph [25, 26]. DA-based reinforcement learning is known to involve plasticity in mammalian cortico-striatal synapses [27], and DA-modulated plasticity of cortical inputs to Area X, measured in brain slice recordings, is observable at intermediate stages of vocal development (47–52 dph) [28, 29], suggesting early innervation of Area X by DA neurons. Importantly, topographic connectivity of cortico-basal ganglia pathways during song system development is affected by manipulations of auditory experience early in life, suggesting that experience can play a role in refining vocal circuitry [30, 31].

This vocal learning process provides a clear timeline for testing the development of the mesostriatal DA systems [32]. By injecting distinctly colored retrograde neuronal tracers into Area X and MST in juvenile birds and examining tissue days later, we tested if VTA cell bodies projected to Area X, MST, or both regions, in the brains of birds that prior to subsong singing (early juveniles, 30–35 dph) and birds that were in the process of learning their distinct song motif (late juveniles, 60–65 dph). By repeating these experiments in adult birds, we tested for the replicability of past studies and compared cell counts in juveniles and adults. If the dopaminergic projections from VTA to MST differentiate based on singing experience, as has been shown in forebrain nuclei of the song system [30], then prior to song learning, single neurons in VTA may project to both MST and Area X. On the other hand, if DA projections from VTA to MST are pre-determined, for example by genetic influences, then VTA neurons will already be segregated by projection area in juveniles. We discovered that the two pathways were completely segregated early in development, supporting the second outcome where a song system-associated DA population exists even before the onset of vocal practice.

## Materials and methods

### Animals

All animal procedures were in accordance with NIH guidelines, the Institutional Animal Care and Use Committee of Cornell University (ID# 2018–0026), and the New York State Department of Health. We used data from 26 healthy male zebra finches (Taeniopygia guttata), age range 30–700 days post-hatch (dph). Birds were obtained from our breeding colonies in Ithaca, New York. Prior to surgery, birds at least 50 dph, were in constant social contact with other males in large colony cages and had visual and auditory contact with females. Birds below 50 dph were in constant social contact with both male and female zebra finches in a large breeding cage. Loosely attached leg bands were used as identifiers for each bird after at least 50 dph. Post-surgery, birds were in individual cages, but had visual and auditory contact with both males and females in separate cages. Perfusion was performed 5 days after surgery. Pre- and post-surgical birds were located in rooms with controlled temperature and humidity conditions on a 12/12 hour day/night schedule with constant access to food and water (seed mix and water refreshed daily by animal technicians employed by Cornell University). Environmental enrichment consisted of mirrors and perches of diverse sizes, and cuttlebones in cages. Weekly enrichment consisted of vegetables, eggs, and water baths.

For surgeries, animals were anesthetized with 2.5% isoflurane and head-fixed on a stereotaxic surgery rig (Leica Biosystems product #39477001) with a 40°C heating pad to help maintain body temperature. Anesthesia was assessed every 5 minutes and isoflurane levels were adjusted between 1–3% according to respiration rate and responsiveness. The scalp was disinfected with 10% Betadine in distilled water and 70% EtOH in distilled water. After craniotomies were made and dura removed, 30 nL of fluorescently labeled cholera toxin subunit B (CTB Alexa 555, Molecular Probes) were injected bilaterally into Area X and 30 nL CTB Alexa 647 (Molecular Probes) was injected into MST bilaterally at the coordinates shown in Table 1. A Nanoject II (Drummond Scientific, Broomall, PA) outfitted with a pulled glass capillary needle was used for each 30 nL injection, consisting of 5 infusions of 9.2 nL each to the designated brain regions with a 10 second wait time between each injection. After all infusions were completed, the needle was left in the brain for 15 minutes so the injected fluid could diffuse before the needle was extracted. Ten minutes prior to the end of surgery, 0.05 mL each of buprenorphine and enrosite were administered via intramuscular injection in the pectoral muscle. A mixture of 5% lidocaine cream and bacitracin antibiotic ointment was applied to the scalp. Following surgery, animals were placed in a small holding cage, recovered quickly, and were typically able to perch, eat and drink within 15 minutes. After recovery, the animals were assessed approximately every hour for 4–6 hours and then assessed at least 3 times a day for 2 days after the surgery.

**Table 1:** Stereotaxic injection coordinates for all surgeries performed on the zebra finches.

	Area X			MST		
	AP	ML	DV	AP	ML	DV
Adult	5.75	1.6	2.85	5.7	0.6	3.1
Late Juveniles	5.65	1.5	2.8	5.7	0.6	3
Early Juveniles	5.65	1.5	2.8	5.5	0.55	3

All injections were carried out using a stereotaxic apparatus (Leica Biosystems) and administered at a 20° head angle, antero-posterior (AP) and mediolateral (ML) coordinates zeroed at the lambda bifurcation, dorso-ventral (DV) coordinates zeroed at the pial surface, and performed bilaterally. Coordinates were determined by test injections performed on zebra finches, following the same injection procedure outlined in the methods with the perfusion method directly following the end of surgery. The brain was sectioned and image processing occurred as described in the methods and histology was compared to the injections from the Person et al. 2008 paper to confirm coordinates.

Surgeries were performed on three age groups, adult birds (>90 dph, n = 12 hemispheres, n = 6 birds), late juveniles (60–65 dph, n = 7 hemispheres, n = 5 birds), and early juveniles (30–35 dph, n = 7 hemispheres, n = 4 birds). For both late juveniles and early juveniles, the anesthesia was adjusted to approximately 1.5% isoflourane, with adjustments between 1–3% according to respiration rate and responsiveness, and 0.03 mL each of buprenorphine and enrosite were administered via intramuscular injection in the pectoral muscle.

### Perfusion and histological processing

Injected birds were perfused transcardially 5 days post-injection with 4% paraformaldehyde (PFA) diluted in phosphate-buffered saline (PBS). The dorsal and caudal portions of the skull were then removed and the whole brain extracted and stored at 4°C for 24–48 hours in PFA. Brains were sliced using a Leica VT1000 S vibratome into 100 m sagittal sections in PBS, mounted on microscope slides under Polyvinyl alcohol mounting medium with DABCO (Sigma Aldrich), and stored at 4°C. All PBS was prepared by diluting 20X Concentrate (VWR Ultra Pure, pH 7.5), 20-fold in deionized water. The 1X PBS used was composed of 135 mM NaCl, 2.7 mM potassium phosphate monobasic anhydrous salt, and 9.42 mM sodium phosphate monobasic.

### Image processing and cell counting

Injection sites were imaged 1–2 days after slicing using a Leica DFC345 FX fluorescent microscope. Along the medial-lateral axis, anatomical landmarks were used to determine the midline of the brain, which, with the constant thickness of each slice, was then used to determine the distance of each slice from the midline. All slices containing portions of VTA and HVC were imaged with both 555 and 647 nm excitation while maintaining the same field of view. Fluorescence in HVC was assessed to confirm Area X injection accuracy, as projections from HVC to Area X are well established in both juvenile and adult zebra finches [31, 33]. MST injection accuracy was assessed with a combination of visual inspection and a lack of fluorescence in HVC (which would indicate diffusion of the injection into Area X because HVC does not project to MST). Images were imported to FIJI ImageJ software for cell counting. Images from the 555 and 647 channels were analyzed individually and in an overlay to count co-labeled cells.

### Statistical analysis

All of the fluorescently labeled cells in VTA in each hemisphere for each age group were counted. To test if age affects expression profiles, we first ran a mixed-effects ANOVA with age, total cell count, hemisphere, and expression (CTB Alexa 555 or 647) as fixed effects, bird ID as the random effect, and raw cell counts as the response variable. Next, we ran an ANOVA for each expression group (CTB Alexa 555 or 647) to test for the possibility of differences among age groups within expression groups. Due to a significant ANOVA F-value for the effect of age on cell count in the MST to VTA-projecting group, a follow up Tukey’s test was performed to assess the significance of the differences between each age group. These tests were repeated for the Area X to VTA-projecting group. Finally, to assess differences in Area X vs MST projecting neurons within age groups, Welch two sample t-tests were performed. To carry out these statistical analyses, we used R programming language (version 4.2.1). Data visualization was performed with both R (version 4.2.1) and MATLAB (2023A).

## Results

### VTA projections to MST or Area X are segregated before the onset of vocal practice

**Fig 1:**
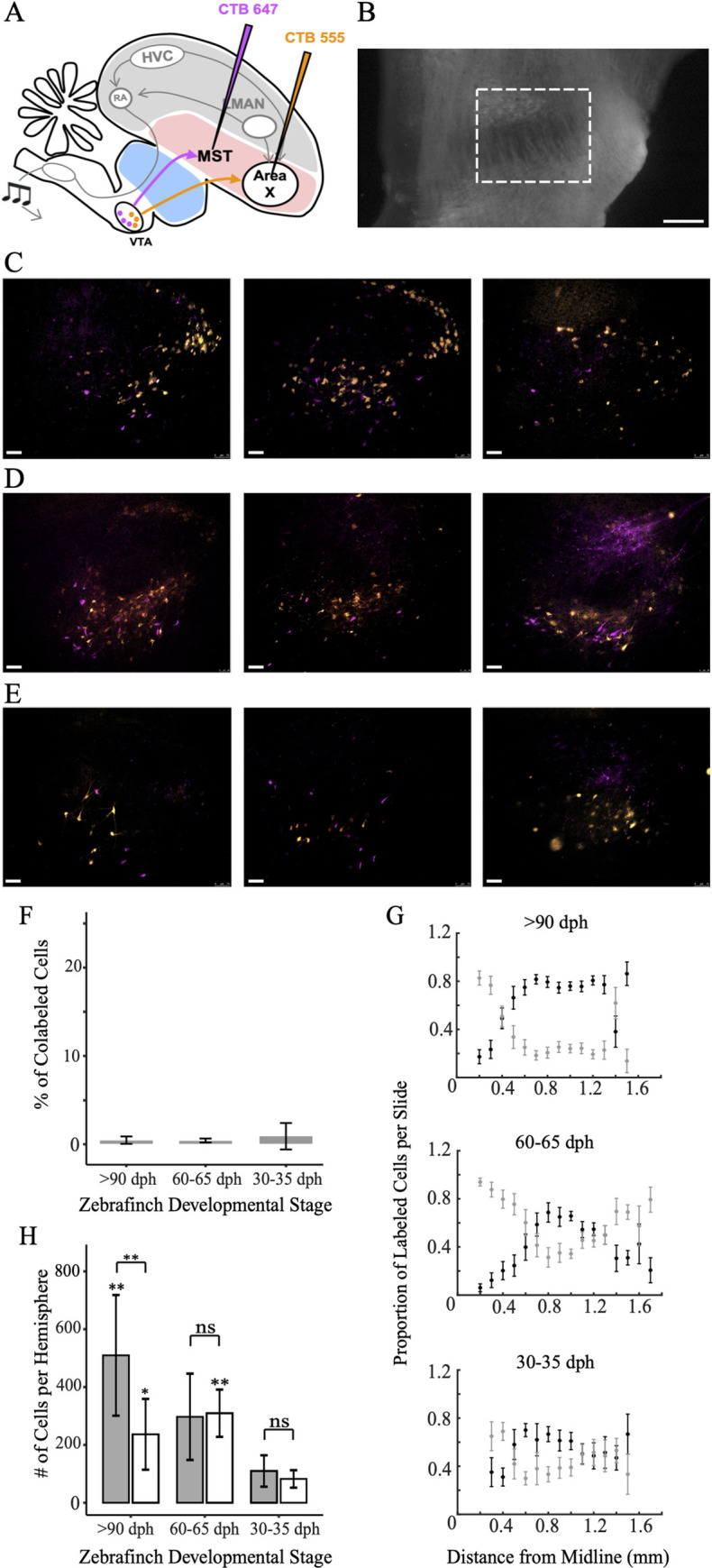
VTA projections to MST or Area X are segregated before the onset of vocal practice. (A) Schematic of the experimental strategy used to label VTA projections to MST and Area X; (B) Darkfield image with expanded view of VTA, denoted by dashed white rectangle, scale bar 250 μm; (C) MST (magenta) and Area X (orange) projecting neurons in VTA in adults. Right to left: examples of a similar region in VTA from three different birds. Scale bar, 75 um. Dorsal is up; anterior is to the right. (D) Same as C except for late juveniles 60–65 dph; (E) Same as C except for early juveniles 30–35 dph. (F) Average cell counts per hemisphere of populations projecting to Area X (dark gray) and MST (white) from VTA in all three injection sites with an n of 12, 7, and 7 hemispheres respectively. Asterisks above vertical error bars for the late juveniles and adult populations show results of Tukey’s post-hoc analysis when comparing to appropriate early juvenile populations. Asterisks above horizontal brackets show results of Welch two sample t-tests between neuron populations within the same age group. (G) Percent of co-labeled cells in VTA that project to both MST and Area X in all three injection sites with an n of 12, 7, and 7 hemispheres respectively. (H) Proportion of Area X (black) and MST (gray) projecting cells over total labeled cells in the brain slice, progressing from medial to lateral. Error bars reflect standard error of the mean. *P < 0.05, **P < 0.01, ***P < 0.001. NS, not significant.

In a previous study using dual-color retrograde tracing experiments with injections into Area X and MST, the VTA_MST_ projection was found to be distinct from the VTA_X_ neuron population in adults, with a colabeling of < 5% [15]. To test if this segregation of DA pathways exists before the onset of vocal learning and examine changes over development, we repeated these experiments in both early juvenile animals (<35 dph, n = 7 hemispheres), intermediate juveniles (60–65 dph, n = 7 hemispheres), and adults (>90 dph, n = 12 hemispheres) (Fig 1A–1E, S1A–S1C Fig). We found that the percent of colabeled VTA_X_ and VTA_MST_ neurons was similarly negligible in adults and in both early and late juvenile groups (11/1346 cells colabeled in 30–35 dph, n = 7 hemispheres; 19/4247 cells colabeled in 60–65 dph, n = 7 hemispheres; 37/8955 cells colabeled in >90 dph, n = 12 hemispheres), with a colabeling of < 1% (Fig 1F). Thus the VTA_MST_ and VTA_X_ pathways are segregated even before the onset of substantial vocal practice.

While adults and juveniles exhibited similarly negligible percentages of colabeled cells, the topographical separation between the two populations widened as juveniles transitioned to adulthood. In early juveniles, the VTA_MST_ and VTA_X_ projections co-mingled in the anterior-dorsal region of VTA (Fig 1G). But in adults and late juveniles, the VTA_MST_ neurons, though still continuous and co-mingled with VTAx neurons, appeared to cluster in two distinct territories: one lateral to the VTA_X_ neurons in dorsal VTA and another medial to the VTA_X_ neurons in the ventral-posterior region of VTA (Fig 1G). These results are compatible with the idea that the VTA_X_ neuronal population expands during development and migrates into the region, displacing MST projectors medially and laterally.

### Outsized expansion of the VTAx population over development

To test the possibility of VTA_X_ population expansion during vocal learning, we examined how the number of VTA_X_ and VTA_MST_ neurons changed over development by comparing cell counts across age groups. A global analysis demonstrated significant differences in VTA_X_ and VTA_MST_ populations among age groups (ANOVA, p = 0.00727). Individual ANOVA tests were performed on the VTA_X_ and VTA_MST_ populations which indicated highly significant changes in cell counts across age groups (VTA_X_ population ANOVA, p = 0.00537; VTA_MST_ population ANOVA, p = 0.012). A follow up Tukey’s test demonstrated significant increases in VTA_X_ and VTA_MST_ populations between early juveniles and adults (VTA_X_ population Tukey, p = 0.0036; VTA_MST_ population Tukey, p = 0.0404). Next, we explored the possibility of diverging counts between VTA_X_ and VTA_MST_ within age groups. In early and late juveniles, the number of VTA_X_ and VTA_MST_ neurons was not significantly different (early: 110±20 VTA_X_ neurons/hemisphere vs 82±11 VTA_MST_ neurons/hemisphere, mean±standard error (SE), Welch two sample t-test, p = 0.27, n = 7 hemispheres; late: 297±56 VTA_X_ neurons/hemisphere vs 309±31 VTA_MST_ neurons/hemisphere, mean±SE, Welch two sample t-test, p = 0.85, n = 7 hemispheres; Fig 1F). However in adults, the number of VTA_X_ neurons dramatically expanded relative to the number of VTA_MST_ neurons (510±60 VTA_X_ neurons/hemisphere vs 237±35 VTA_MST_ neurons/hemisphere, mean±SE, Welch two sample t-test, p = 0.001, n = 12 hemispheres; Fig 1F). These results indicate an outsized growth of the VTA_X_ projection over the same developmental period when the bird is learning its song.

## Discussion

Distinct pathways within the larger mesostriatal DA system that carry different types of reinforcement signals could enable animals to learn in complex conditions and across a wide range of objectives [1, 9, 17, 34–37]. Recent anatomical and physiological studies show that segregated pathways indeed exist and can carry distinct types of signals [7–10, 38, 39]. These studies raise the question of how, and when, segregation within the mesostriatal system is formed. Past work in mammals showed that dopaminergic innervation of patch striatal areas precede matrix ones during embryogenesis [40], but it remains unclear how these results map to functionally distinct circuits that may also be spatially segregated, as in the songbird [14, 15]. Here we focused on two segregated channels in the songbird to determine the role of development and experience on DA pathway formation.

The VTA projection to the song system striatal nucleus Area X carries singing related error signals, controls striatal plasticity, and is important for song learning [16, 17, 19, 20]. A DA projection to an adjacent striatal nucleus, MST, arises from a distinct group of neurons that reside in an overlapping part of VTA, and we confirmed that the VTA_X_ and VTA_MST_ populations were distinct in adults [14, 15]. The key novel finding of the present manuscript is that this spatial segregation also exists in very young birds (<35 dph) that have had little, if any, vocal practice. These results support a model where the segregation of these pathways is genetically determined, though we cannot rule out the possibility that even earlier life experience, for example during begging calls, may play a role [41]. Future studies in even younger birds (~10 dph) would be necessary to address this question, but due to the technical difficulties of survival neurosurgeries in bird embryos, genetic methods may need to be deployed.

We also discovered that the number of VTA_X_ neurons expands during the time course of vocal development. Past work shows that DA modulated plasticity exists in Area X during vocal development, around the same time we observe growth in the DA input to Area X [28, 29]. These data suggest that, during the key period of vocal development in zebra finches, the VTA to Area X projection significantly increases. It is also possible that these changes are due to altered ability of the retrograde tracer to be taken up and transported, which would occur if there was outsized expansion in the arborization of VTA axons in Area X. Substantial remodeling, including invasion of axons into other song system nuclei, have also been reported during this timeframe [14, 30]. We also noted that the VTA_MST_ neuron population expands during the time course of vocal development, but the population sizes of VTA_MST_ and VTA_X_ neurons differ past 65 dph with the VTA_X_ neurons outnumbering VTA_MST_ neurons on average more than two to one in adults (Fig 1F).

If findings from the avian system generalize to mammals, our data would suggest distinct mesostriatal systems in mammals, for example that carry aversive versus reinforcing outcome signals [8, 10, 38, 39], may be patterned very early in life, even before valent experiences [40, 42]. Future studies in mammals examining mesostriatal development in pathways known to exhibit distinct types of outcome signals will be important to test for genetic versus experience-dependent modeling of the mesostriatal system.

## Supporting information

S1 Fig Confirmation of Area X and MST injections. (A-C) Area X, denoted by dashed white lines, (left) and MST (right) injections. Insets show HVC, denoted by dashed white lines. Note retrogradely labeled HVC neurons following Area X injections and absence of neurons following MST injections, confirming absence of leakage from MST injections into Area X. (A) adults; (B) late juveniles; (C) early juveniles. Scale bars are 250 μm in injection images and 50 μm in inset images.
(TIF)

S1 Table Raw data cell counts for all birds. All counted cells in VTA projecting to Area X, MST, and that are co-labeled with n = 12, n = 7, and n = 7 hemispheres for adult, late juvenile, and early juvenile injection sites respectively.
(DOCX)

S2 Table Raw data cell counts per slide for each bird. All counted cells in VTA projecting to Area X or MST based on slide number that was matched to the distance from the midline. The procedure used to determine the slide number and distance from the midline were listed in the methods section. Additionally, please note that any slides without any Area X or MST projections were omitted from the tables.
(DOCX)

S3 Table Proportion of labeled cells per slide and standard error. Using the procedure described in the methods section above, the proportion of labeled cells per slide and standard error was calculated for each age group. The final values used to construct Fig 1H are included in the tables below. Standard error is abbreviated to SE, the proportion of Area X labeled cells per slide is abbreviated to PX, and the proportion of MST labeled cells per slide is abbreviated to PMST.
(DOCX)
