# Supplementary figures and images for: Songbird mesostriatal dopamine pathways are spatially segregated before the onset of vocal learning
Source: PLoS One. 2023 Nov 16;18(11):e0285652. doi: 10.1371/journal.pone.0285652 (PMC10653429; doi:10.1371/journal.pone.0285652)

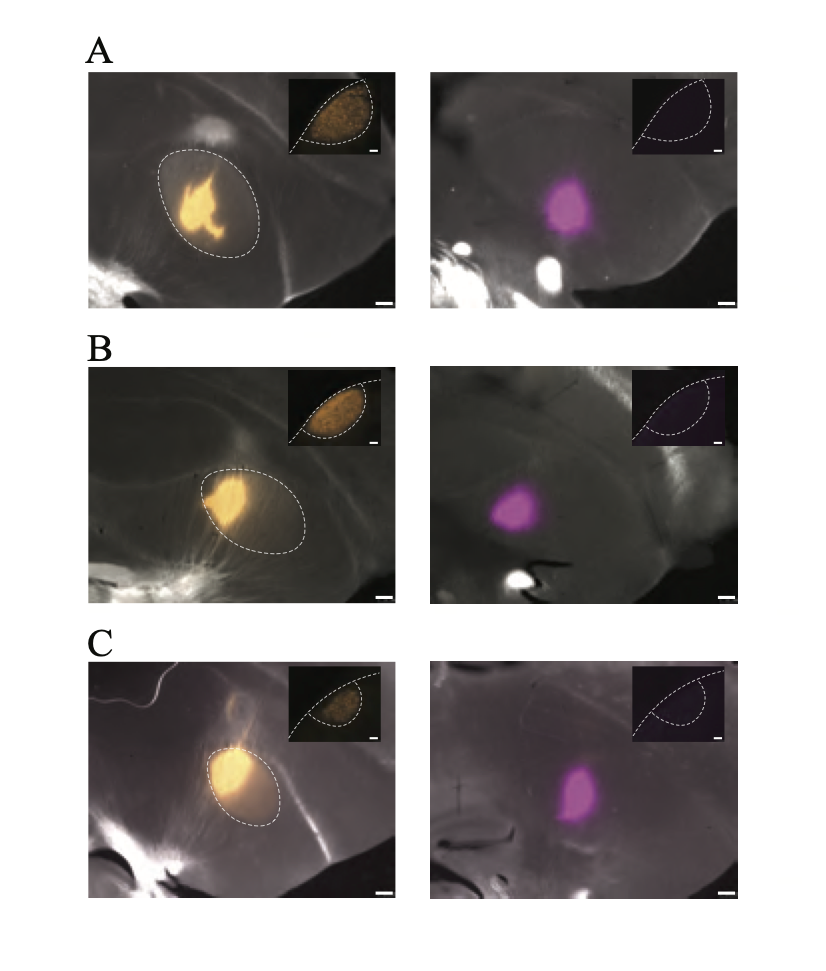

Supplement: S1 Fig — (A-C) Area X, denoted by dashed white lines, (left) and MST (right) injections. Insets show HVC, denoted by dashed white lines. Note retrogradely labeled HVC neurons following Area X injections and absence of neurons following MST injections, confirming absence of leakage from MST injections into Area X. (A) adults; (B) late juveniles; (C) early juveniles. Scale bars are 250 μm in injection images and 50 μm in inset images. (TIF) [file pone.0285652.s001.tif]
