# Supplementary material for: Songbird mesostriatal dopamine pathways are spatially segregated before the onset of vocal learning
Source: PLoS One. 2023 Nov 16;18(11):e0285652. doi: 10.1371/journal.pone.0285652 (PMC10653429; doi:10.1371/journal.pone.0285652)
Supplement: S1 Table — All counted cells in VTA projecting to Area X, MST, and that are co-labeled with n = 12, n = 7, and n = 7 hemispheres for adult, late juvenile, and early juvenile injection sites respectively. (DOCX) [file pone.0285652.s002.docx]

**Supplementary Table 1: Raw Data Cell Counts for All Birds**

All counted cells in VTA projecting to Area X, MST, and that are co-labeled with n = 12, n = 7, and n = 7 hemispheres for adult, late juvenile, and early juvenile injection sites respectively.

| **Bird ID** | **Injection Site** | **AreaX Projections** | **MST Projections** | **Colabeled Cells** |
| --- | --- | --- | --- | --- |
| Adult1_RH | Adult | 701 | 99 | 10 |
| Adult1_LH | Adult | 430 | 189 | 6 |
| Adult2_RH | Adult | 369 | 258 | 1 |
| Adult2_LH | Adult | 143 | 251 | 3 |
| Adult3_RH | Adult | 660 | 439 | 1 |
| Adult3_LH | Adult | 524 | 218 | 6 |
| Adult4_RH | Adult | 252 | 148 | 3 |
| Adult4_LH | Adult | 369 | 103 | 1 |
| Adult5_RH | Adult | 548 | 107 | 0 |
| Adult5_LH | Adult | 543 | 221 | 1 |
| Adult6_RH | Adult | 873 | 445 | 1 |
| Adult6_LH | Adult | 704 | 361 | 4 |
| LJuv1_LH | Late Juvenile | 48 | 194 | 0 |
| LJuv2_RH | Late Juvenile | 404 | 423 | 4 |
| LJuv2_LH | Late Juvenile | 303 | 327 | 2 |
| LJuv3_RH | Late Juvenile | 526 | 397 | 3 |
| LJuv4_LH | Late Juvenile | 311 | 312 | 4 |
| LJuv5_RH | Late Juvenile | 214 | 270 | 3 |
| LJuv5_LH | Late Juvenile | 274 | 244 | 3 |
| EJuv1_RH | Early Juvenile | 82 | 97 | 7 |
| EJuv1_LH | Early Juvenile | 79 | 79 | 3 |
| EJuv2_RH | Early Juvenile | 29 | 50 | 0 |
| EJuv2_LH | Early Juvenile | 161 | 52 | 0 |
| EJuv3_RH | Early Juvenile | 192 | 138 | 0 |
| EJuv3_LH | Early Juvenile | 112 | 90 | 0 |
| EJuv4_RH | Early Juvenile | 114 | 71 | 1 |
