# Supplementary material for: Songbird mesostriatal dopamine pathways are spatially segregated before the onset of vocal learning
Source: PLoS One. 2023 Nov 16;18(11):e0285652. doi: 10.1371/journal.pone.0285652 (PMC10653429; doi:10.1371/journal.pone.0285652)
Supplement: S2 Table — All counted cells in VTA projecting to Area X or MST based on slide number that was matched to the distance from the midline. The procedure used to determine the slide number and distance from the midline were listed in the methods section. Additionally, please note that any slides without any Area X or MST projections were omitted from the tables. (DOCX) [file pone.0285652.s003.docx]

**Supplementary Table 2: Raw Data Cell Counts Per Slide for Each Bird**

| **Bird ID** | **Distance from Midline (mm)** | **Slide Number** | **Area X Projections** | **MST Projections** |
| --- | --- | --- | --- | --- |
| Adult1_RH | 1.5 | 2 | 1 | 0 |
|  | 1.4 | 3 | 0 | 0 |
|  | 1.3 | 4 | 18 | 0 |
|  | 1.2 | 5 | 47 | 0 |
|  | 1.1 | 6 | 92 | 0 |
|  | 1 | 7 | 96 | 6 |
|  | 0.9 | 8 | 99 | 16 |
|  | 0.8 | 9 | 104 | 13 |
|  | 0.7 | 10 | 69 | 15 |
|  | 0.6 | 11 | 72 | 19 |
|  | 0.5 | 12 | 103 | 30 |

| **Bird ID** | **Distance from Midline (mm)** | **Slide Number** | **Area X Projections** | **MST Projections** |
| --- | --- | --- | --- | --- |
| Adult1_LH | 1.5 | 2 | 6 | 0 |
|  | 1.4 | 3 | 14 | 7 |
|  | 1.3 | 4 | 36 | 2 |
|  | 1.2 | 5 | 37 | 6 |
|  | 1.1 | 6 | 42 | 7 |
|  | 1 | 7 | 33 | 11 |
|  | 0.9 | 8 | 18 | 4 |
|  | 0.8 | 9 | 25 | 3 |
|  | 0.7 | 10 | 52 | 14 |
|  | 0.6 | 11 | 79 | 23 |
|  | 0.5 | 12 | 74 | 34 |
|  | 0.4 | 13 | 14 | 41 |
|  | 0.3 | 14 | 0 | 37 |

| **Bird ID** | **Distance from Midline (mm)** | **Slide Number** | **Area X Projections** | **MST Projections** |
| --- | --- | --- | --- | --- |
| Adult2_RH | 1.4 | 3 | 1 | 9 |
|  | 1.3 | 4 | 3 | 8 |
|  | 1.2 | 5 | 16 | 2 |
|  | 1.1 | 6 | 19 | 18 |
|  | 1 | 7 | 39 | 17 |
|  | 0.9 | 8 | 45 | 29 |
|  | 0.8 | 9 | 41 | 23 |
|  | 0.7 | 10 | 49 | 14 |
|  | 0.6 | 11 | 37 | 16 |
|  | 0.5 | 12 | 51 | 14 |
|  | 0.4 | 13 | 37 | 27 |
|  | 0.3 | 14 | 22 | 49 |
|  | 0.2 | 15 | 9 | 27 |

| **Bird ID** | **Distance from Midline (mm)** | **Slide Number** | **Area X Projections** | **MST Projections** |
| --- | --- | --- | --- | --- |
| Adult2_LH | 1.5 | 2 | 3 | 0 |
|  | 1.4 | 3 | 0 | 1 |
|  | 1.3 | 4 | 13 | 10 |
|  | 1.2 | 5 | 17 | 12 |
|  | 1.1 | 6 | 16 | 16 |
|  | 1 | 7 | 22 | 17 |
|  | 0.9 | 8 | 13 | 18 |
|  | 0.8 | 9 | 13 | 17 |
|  | 0.7 | 10 | 27 | 12 |
|  | 0.6 | 11 | 13 | 26 |
|  | 0.5 | 12 | 2 | 35 |
|  | 0.4 | 13 | 2 | 48 |
|  | 0.3 | 14 | 2 | 34 |
|  | 0.2 | 15 | 0 | 5 |

| **Bird ID** | **Distance from Midline (mm)** | **Slide Number** | **Area X Projections** | **MST Projections** |
| --- | --- | --- | --- | --- |
| Adult3_RH | 1.4 | 3 | 0 | 1 |
|  | 1.3 | 4 | 9 | 5 |
|  | 1.2 | 5 | 26 | 13 |
|  | 1.1 | 6 | 55 | 24 |
|  | 1 | 7 | 51 | 38 |
|  | 0.9 | 8 | 42 | 28 |
|  | 0.8 | 9 | 41 | 3 |
|  | 0.7 | 10 | 51 | 9 |
|  | 0.6 | 11 | 124 | 9 |
|  | 0.5 | 12 | 127 | 16 |
|  | 0.4 | 13 | 76 | 12 |

| **Bird ID** | **Distance from Midline (mm)** | **Slide Number** | **Area X Projections** | **MST Projections** |
| --- | --- | --- | --- | --- |
| Adult3_LH | 1.3 | 4 | 7 | 0 |
|  | 1.2 | 5 | 20 | 10 |
|  | 1.1 | 6 | 36 | 20 |
|  | 1 | 7 | 51 | 19 |
|  | 0.9 | 8 | 38 | 10 |
|  | 0.8 | 9 | 22 | 4 |
|  | 0.7 | 10 | 36 | 2 |
|  | 0.6 | 11 | 104 | 6 |
|  | 0.5 | 12 | 107 | 15 |
|  | 0.4 | 13 | 70 | 38 |
|  | 0.3 | 14 | 28 | 27 |
|  | 0.2 | 15 | 5 | 19 |

| **Bird ID** | **Distance from Midline (mm)** | **Slide Number** | **Area X Projections** | **MST Projections** |
| --- | --- | --- | --- | --- |
| Adult4_RH | 1.2 | 5 | 4 | 0 |
|  | 1.1 | 6 | 13 | 3 |
|  | 1 | 7 | 33 | 7 |
|  | 0.9 | 8 | 11 | 6 |
|  | 0.8 | 9 | 36 | 9 |
|  | 0.7 | 10 | 25 | 9 |
|  | 0.6 | 11 | 35 | 11 |
|  | 0.5 | 12 | 67 | 56 |
|  | 0.4 | 13 | 25 | 33 |
|  | 0.3 | 14 | 3 | 14 |
|  | 0.2 | 15 | 0 | 0 |

| **Bird ID** | **Distance from Midline (mm)** | **Slide Number** | **Area X Projections** | **MST Projections** |
| --- | --- | --- | --- | --- |
| Adult4_LH | 1.4 | 3 | 3 | 2 |
|  | 1.3 | 4 | 5 | 0 |
|  | 1.2 | 5 | 16 | 7 |
|  | 1.1 | 6 | 21 | 10 |
|  | 1 | 7 | 42 | 14 |
|  | 0.9 | 8 | 40 | 4 |
|  | 0.8 | 9 | 34 | 11 |
|  | 0.7 | 10 | 54 | 8 |
|  | 0.6 | 11 | 92 | 13 |
|  | 0.5 | 12 | 51 | 13 |
|  | 0.4 | 13 | 11 | 9 |
|  | 0.3 | 14 | 0 | 12 |

| **Bird ID** | **Distance from Midline (mm)** | **Slide Number** | **Area X Projections** | **MST Projections** |
| --- | --- | --- | --- | --- |
| Adult5_RH | 1.4 | 3 | 20 | 2 |
|  | 1.3 | 4 | 84 | 11 |
|  | 1.2 | 5 | 56 | 10 |
|  | 1.1 | 6 | 81 | 16 |
|  | 1 | 7 | 66 | 19 |
|  | 0.9 | 8 | 53 | 22 |
|  | 0.8 | 9 | 53 | 9 |
|  | 0.7 | 10 | 97 | 5 |
|  | 0.6 | 11 | 38 | 13 |

| **Bird ID** | **Distance from Midline (mm)** | **Slide Number** | **Area X Projections** | **MST Projections** |
| --- | --- | --- | --- | --- |
| Adult5_LH | 1.4 | 3 | 0 | 2 |
|  | 1.3 | 4 | 40 | 7 |
|  | 1.2 | 5 | 75 | 19 |
|  | 1.1 | 6 | 73 | 12 |
|  | 1 | 7 | 40 | 2 |
|  | 0.9 | 8 | 50 | 2 |
|  | 0.8 | 9 | 45 | 1 |
|  | 0.7 | 10 | 54 | 1 |
|  | 0.6 | 11 | 108 | 3 |
|  | 0.5 | 12 | 45 | 2 |
|  | 0.4 | 13 | 10 | 21 |
|  | 0.3 | 14 | 2 | 5 |

| **Bird ID** | **Distance from Midline (mm)** | **Slide Number** | **Area X Projections** | **MST Projections** |
| --- | --- | --- | --- | --- |
| Adult6_RH | 1.6 | 1 | 0 | 1 |
|  | 1.5 | 2 | 2 | 2 |
|  | 1.4 | 3 | 3 | 9 |
|  | 1.3 | 4 | 10 | 12 |
|  | 1.2 | 5 | 39 | 8 |
|  | 1.1 | 6 | 87 | 22 |
|  | 1 | 7 | 68 | 29 |
|  | 0.9 | 8 | 85 | 27 |
|  | 0.8 | 9 | 85 | 11 |
|  | 0.7 | 10 | 71 | 9 |
|  | 0.6 | 11 | 89 | 13 |
|  | 0.5 | 12 | 122 | 22 |
|  | 0.4 | 13 | 115 | 40 |
|  | 0.3 | 14 | 71 | 63 |
|  | 0.2 | 15 | 20 | 66 |

| **Bird ID** | **Distance from Midline (mm)** | **Slide Number** | **Area X Projections** | **MST Projections** |
| --- | --- | --- | --- | --- |
| Adult6_LH | 1.6 | 1 | 0 | 0 |
|  | 1.5 | 2 | 22 | 5 |
|  | 1.4 | 3 | 38 | 4 |
|  | 1.3 | 4 | 49 | 7 |
|  | 1.2 | 5 | 59 | 11 |
|  | 1.1 | 6 | 77 | 9 |
|  | 1 | 7 | 85 | 14 |
|  | 0.9 | 8 | 139 | 19 |
|  | 0.8 | 9 | 111 | 68 |
|  | 0.7 | 10 | 83 | 85 |
|  | 0.6 | 11 | 36 | 87 |
|  | 0.5 | 12 | 5 | 52 |
|  | 0.4 | 13 | 0 | 0 |

| **Bird ID** | **Distance from Midline (mm)** | **Slide Number** | **Area X Projections** | **MST Projections** |
| --- | --- | --- | --- | --- |
| LJuv1_LH | 1.5 | 3 | 1 | 3 |
|  | 1.4 | 4 | 2 | 8 |
|  | 1.3 | 5 | 3 | 17 |
|  | 1.2 | 6 | 10 | 22 |
|  | 1.1 | 7 | 5 | 21 |
|  | 1 | 8 | 11 | 9 |
|  | 0.9 | 9 | 3 | 6 |
|  | 0.8 | 10 | 9 | 9 |
|  | 0.7 | 11 | 3 | 23 |
|  | 0.6 | 12 | 1 | 37 |
|  | 0.5 | 13 | 0 | 39 |

| **Bird ID** | **Distance from Midline (mm)** | **Slide Number** | **Area X Projections** | **MST Projections** |
| --- | --- | --- | --- | --- |
| LJuv2_RH | 1.7 | 1 | 1 | 2 |
|  | 1.6 | 2 | 4 | 1 |
|  | 1.5 | 3 | 1 | 8 |
|  | 1.4 | 4 | 0 | 11 |
|  | 1.3 | 5 | 22 | 16 |
|  | 1.2 | 6 | 32 | 23 |
|  | 1.1 | 7 | 31 | 25 |
|  | 1 | 8 | 39 | 17 |
|  | 0.9 | 9 | 45 | 13 |
|  | 0.8 | 10 | 44 | 23 |
|  | 0.7 | 11 | 89 | 41 |
|  | 0.6 | 12 | 56 | 82 |
|  | 0.5 | 13 | 29 | 94 |
|  | 0.4 | 14 | 10 | 46 |
|  | 0.3 | 15 | 1 | 21 |

| **Bird ID** | **Distance from Midline (mm)** | **Slide Number** | **Area X Projections** | **MST Projections** |
| --- | --- | --- | --- | --- |
| LJuv2_LH | 1.4 | 4 | 3 | 5 |
|  | 1.3 | 5 | 14 | 9 |
|  | 1.2 | 6 | 13 | 16 |
|  | 1.1 | 7 | 13 | 12 |
|  | 1 | 8 | 21 | 16 |
|  | 0.9 | 9 | 39 | 10 |
|  | 0.8 | 10 | 52 | 8 |
|  | 0.7 | 11 | 67 | 27 |
|  | 0.6 | 12 | 62 | 70 |
|  | 0.5 | 13 | 15 | 60 |
|  | 0.4 | 14 | 2 | 46 |
|  | 0.3 | 15 | 0 | 17 |
|  | 0.2 | 16 | 0 | 10 |

| **Bird ID** | **Distance from Midline (mm)** | **Slide Number** | **Area X Projections** | **MST Projections** |
| --- | --- | --- | --- | --- |
| LJuv3_RH | 1.7 | 1 | 0 | 2 |
|  | 1.6 | 2 | 0 | 2 |
|  | 1.5 | 3 | 1 | 2 |
|  | 1.4 | 4 | 0 | 6 |
|  | 1.3 | 5 | 6 | 13 |
|  | 1.2 | 6 | 21 | 14 |
|  | 1.1 | 7 | 51 | 31 |
|  | 1 | 8 | 63 | 23 |
|  | 0.9 | 9 | 48 | 10 |
|  | 0.8 | 10 | 22 | 2 |
|  | 0.7 | 11 | 39 | 4 |
|  | 0.6 | 12 | 104 | 8 |
|  | 0.5 | 13 | 93 | 45 |
|  | 0.4 | 14 | 52 | 77 |
|  | 0.3 | 15 | 23 | 66 |
|  | 0.2 | 16 | 3 | 65 |

| **Bird ID** | **Distance from Midline (mm)** | **Slide Number** | **Area X Projections** | **MST Projections** |
| --- | --- | --- | --- | --- |
| LJuv4_LH | 1.7 | 1 | 2 | 5 |
|  | 1.6 | 2 | 2 | 2 |
|  | 1.5 | 3 | 5 | 6 |
|  | 1.4 | 4 | 8 | 13 |
|  | 1.3 | 5 | 21 | 23 |
|  | 1.2 | 6 | 38 | 26 |
|  | 1.1 | 7 | 20 | 18 |
|  | 1 | 8 | 20 | 4 |
|  | 0.9 | 9 | 17 | 5 |
|  | 0.8 | 10 | 63 | 5 |
|  | 0.7 | 11 | 72 | 39 |
|  | 0.6 | 12 | 30 | 59 |
|  | 0.5 | 13 | 9 | 33 |
|  | 0.4 | 14 | 1 | 26 |
|  | 0.3 | 15 | 1 | 37 |
|  | 0.2 | 16 | 2 | 11 |

| **Bird ID** | **Distance from Midline (mm)** | **Slide Number** | **Area X Projections** | **MST Projections** |
| --- | --- | --- | --- | --- |
| LJuv5_LH | 1.6 | 2 | 2 | 3 |
|  | 1.5 | 3 | 2 | 3 |
|  | 1.4 | 4 | 11 | 2 |
|  | 1.3 | 5 | 18 | 6 |
|  | 1.2 | 6 | 59 | 18 |
|  | 1.1 | 7 | 52 | 16 |
|  | 1 | 8 | 53 | 36 |
|  | 0.9 | 9 | 30 | 50 |
|  | 0.8 | 10 | 27 | 28 |
|  | 0.7 | 11 | 16 | 24 |
|  | 0.6 | 12 | 3 | 17 |
|  | 0.5 | 13 | 0 | 14 |
|  | 0.4 | 14 | 1 | 9 |

| **Bird ID** | **Distance from Midline (mm)** | **Slide Number** | **Area X Projections** | **MST Projections** |
| --- | --- | --- | --- | --- |
| LJuv5_RH | 1.4 | 4 | 1 | 2 |
|  | 1.3 | 5 | 8 | 5 |
|  | 1.2 | 6 | 29 | 26 |
|  | 1.1 | 7 | 36 | 21 |
|  | 1 | 8 | 23 | 14 |
|  | 0.9 | 9 | 18 | 9 |
|  | 0.8 | 10 | 9 | 11 |
|  | 0.7 | 11 | 12 | 7 |
|  | 0.6 | 12 | 24 | 27 |
|  | 0.5 | 13 | 25 | 39 |
|  | 0.4 | 14 | 16 | 19 |
|  | 0.3 | 15 | 10 | 25 |
|  | 0.2 | 16 | 3 | 65 |

| **Bird ID** | **Distance from Midline (mm)** | **Slide Number** | **Area X Projections** | **MST Projections** |
| --- | --- | --- | --- | --- |
| EJuv1_RH | 1.5 | 2 | 6 | 6 |
|  | 1.4 | 3 | 1 | 5 |
|  | 1.3 | 4 | 5 | 11 |
|  | 1.2 | 5 | 12 | 12 |
|  | 1.1 | 6 | 14 | 26 |
|  | 1 | 7 | 23 | 23 |
|  | 0.9 | 8 | 12 | 9 |
|  | 0.8 | 9 | 9 | 4 |
|  | 0.7 | 10 | 0 | 1 |

| **Bird ID** | **Distance from Midline (mm)** | **Slide Number** | **Area X Projections** | **MST Projections** |
| --- | --- | --- | --- | --- |
| EJuv1_LH | 1.6 | 1 | 3 | 4 |
|  | 1.5 | 2 | 0 | 0 |
|  | 1.4 | 3 | 5 | 15 |
|  | 1.3 | 4 | 6 | 18 |
|  | 1.2 | 5 | 20 | 17 |
|  | 1.1 | 6 | 13 | 11 |
|  | 1 | 7 | 17 | 8 |
|  | 0.9 | 8 | 9 | 2 |
|  | 0.8 | 9 | 5 | 3 |
|  | 0.7 | 10 | 1 | 1 |

| **Bird ID** | **Distance from Midline (mm)** | **Slide Number** | **Area X Projections** | **MST Projections** |
| --- | --- | --- | --- | --- |
| EJuv2_RH | 1.4 | 3 | 1 | 1 |
|  | 1.3 | 4 | 0 | 2 |
|  | 1.2 | 5 | 1 | 4 |
|  | 1.1 | 6 | 6 | 14 |
|  | 1 | 7 | 16 | 13 |
|  | 0.9 | 8 | 3 | 12 |
|  | 0.8 | 9 | 2 | 4 |

| **Bird ID** | **Distance from Midline (mm)** | **Slide Number** | **Area X Projections** | **MST Projections** |
| --- | --- | --- | --- | --- |
| EJuv2_LH | 1.5 | 2 | 1 | 1 |
|  | 1.4 | 3 | 4 | 1 |
|  | 1.3 | 4 | 8 | 5 |
|  | 1.2 | 5 | 31 | 13 |
|  | 1.1 | 6 | 24 | 9 |
|  | 1 | 7 | 26 | 6 |
|  | 0.9 | 8 | 11 | 1 |
|  | 0.8 | 9 | 13 | 2 |
|  | 0.7 | 10 | 20 | 5 |
|  | 0.6 | 11 | 16 | 4 |
|  | 0.5 | 12 | 7 | 5 |

| **Bird ID** | **Distance from Midline (mm)** | **Slide Number** | **Area X Projections** | **MST Projections** |
| --- | --- | --- | --- | --- |
| EJuv4_RH | 1.3 | 4 | 1 | 0 |
|  | 1.2 | 5 | 0 | 2 |
|  | 1.1 | 6 | 2 | 6 |
|  | 1 | 7 | 20 | 3 |
|  | 0.9 | 8 | 17 | 10 |
|  | 0.8 | 9 | 20 | 6 |
|  | 0.7 | 10 | 21 | 5 |
|  | 0.6 | 11 | 19 | 13 |
|  | 0.5 | 12 | 11 | 11 |
|  | 0.4 | 13 | 3 | 15 |

| **Bird ID** | **Distance from Midline (mm)** | **Slide Number** | **Area X Projections** | **MST Projections** |
| --- | --- | --- | --- | --- |
| EJuv3_RH | 1.5 | 2 | 1 | 0 |
|  | 1.4 | 3 | 7 | 3 |
|  | 1.3 | 4 | 10 | 7 |
|  | 1.2 | 5 | 19 | 5 |
|  | 1.1 | 6 | 28 | 5 |
|  | 1 | 7 | 13 | 12 |
|  | 0.9 | 8 | 21 | 18 |
|  | 0.8 | 9 | 10 | 4 |
|  | 0.7 | 10 | 26 | 6 |
|  | 0.6 | 11 | 15 | 4 |
|  | 0.5 | 12 | 22 | 2 |
|  | 0.4 | 13 | 8 | 15 |
|  | 0.3 | 14 | 8 | 9 |
|  | 0.2 | 15 | 0 | 26 |

| **Bird ID** | **Distance from Midline (mm)** | **Slide Number** | **Area X Projections** | **MST Projections** |
| --- | --- | --- | --- | --- |
| EJuv3_LH | 1.4 | 3 | 2 | 3 |
|  | 1.3 | 4 | 10 | 2 |
|  | 1.2 | 5 | 10 | 5 |
|  | 1.1 | 6 | 3 | 3 |
|  | 1 | 7 | 2 | 4 |
|  | 0.9 | 8 | 18 | 11 |
|  | 0.8 | 9 | 12 | 6 |
|  | 0.7 | 10 | 20 | 5 |
|  | 0.6 | 11 | 13 | 8 |
|  | 0.5 | 12 | 9 | 19 |
|  | 0.4 | 13 | 10 | 14 |
|  | 0.3 | 14 | 3 | 10 |

All counted cells in VTA projecting to Area X or MST based on slide number that was matched to the distance from the midline. The procedure used to determine the slide number and distance from the midline were listed in the methods section. Additionally, please note that any slides without any Area X or MST projections were omitted from the tables.
