# Supplementary material for: Songbird mesostriatal dopamine pathways are spatially segregated before the onset of vocal learning
Source: PLoS One. 2023 Nov 16;18(11):e0285652. doi: 10.1371/journal.pone.0285652 (PMC10653429; doi:10.1371/journal.pone.0285652)
Supplement: S3 Table — Using the procedure described in the methods section above, the proportion of labeled cells per slide and standard error was calculated for each age group. The final values used to construct Fig 1H are included in the tables below. Standard error is abbreviated to SE, the proportion of Area X labeled cells per slide is abbreviated to PX, and the proportion of MST labeled cells per slide is abbreviated to PMST. (DOCX) [file pone.0285652.s004.docx]

**Supplementary Table 3: Proportion of Labeled Cells per Slide and Standard Error**

| **Age Group** | **Distance from Midline (mm)** | **PX** | **PMST** | **SE of PX** | **SE of PMST** |
| --- | --- | --- | --- | --- | --- |
| Adults | 1.5 | 0.86 | 0.14 | 0.10 | 0.10 |
|  | 1.4 | 0.38 | 0.62 | 0.13 | 0.13 |
|  | 1.3 | 0.77 | 0.23 | 0.08 | 0.08 |
|  | 1.2 | 0.81 | 0.19 | 0.04 | 0.04 |
|  | 1.1 | 0.76 | 0.24 | 0.04 | 0.04 |
|  | 1 | 0.76 | 0.24 | 0.04 | 0.04 |
|  | 0.9 | 0.75 | 0.25 | 0.05 | 0.05 |
|  | 0.8 | 0.79 | 0.21 | 0.05 | 0.05 |
|  | 0.7 | 0.82 | 0.18 | 0.04 | 0.04 |
|  | 0.6 | 0.75 | 0.25 | 0.06 | 0.06 |
|  | 0.5 | 0.66 | 0.34 | 0.09 | 0.09 |
|  | 0.4 | 0.49 | 0.51 | 0.09 | 0.09 |
|  | 0.3 | 0.23 | 0.77 | 0.08 | 0.08 |
|  | 0.2 | 0.17 | 0.83 | 0.06 | 0.06 |

| **Age Group** | **Distance from Midline (mm)** | **PX** | **PMST** | **SE of PX** | **SE of PMST** |
| --- | --- | --- | --- | --- | --- |
| Late Juveniles | 1.7 | 0.21 | 0.79 | 0.10 | 0.10 |
|  | 1.6 | 0.43 | 0.58 | 0.17 | 0.17 |
|  | 1.5 | 0.31 | 0.69 | 0.06 | 0.06 |
|  | 1.4 | 0.31 | 0.69 | 0.11 | 0.11 |
|  | 1.3 | 0.50 | 0.50 | 0.08 | 0.08 |
|  | 1.2 | 0.55 | 0.45 | 0.05 | 0.05 |
|  | 1.1 | 0.54 | 0.46 | 0.07 | 0.07 |
|  | 1 | 0.66 | 0.34 | 0.04 | 0.04 |
|  | 0.9 | 0.65 | 0.35 | 0.08 | 0.08 |
|  | 0.8 | 0.69 | 0.31 | 0.08 | 0.08 |
|  | 0.7 | 0.59 | 0.41 | 0.10 | 0.10 |
|  | 0.6 | 0.40 | 0.60 | 0.11 | 0.11 |
|  | 0.5 | 0.24 | 0.76 | 0.09 | 0.09 |
|  | 0.4 | 0.20 | 0.80 | 0.08 | 0.08 |
|  | 0.3 | 0.12 | 0.88 | 0.06 | 0.06 |
|  | 0.2 | 0.06 | 0.94 | 0.03 | 0.03 |

| **Age Group** | **Distance from Midline (mm)** | **PX** | **PMST** | **SE of PX** | **SE of PMST** |
| --- | --- | --- | --- | --- | --- |
| Early Juveniles | 1.5 | 0.67 | 0.33 | 0.17 | 0.17 |
|  | 1.4 | 0.47 | 0.53 | 0.10 | 0.10 |
|  | 1.3 | 0.51 | 0.49 | 0.13 | 0.13 |
|  | 1.2 | 0.49 | 0.51 | 0.11 | 0.11 |
|  | 1.1 | 0.50 | 0.50 | 0.08 | 0.08 |
|  | 1 | 0.61 | 0.39 | 0.07 | 0.07 |
|  | 0.9 | 0.61 | 0.39 | 0.09 | 0.09 |
|  | 0.8 | 0.67 | 0.33 | 0.06 | 0.06 |
|  | 0.7 | 0.62 | 0.38 | 0.13 | 0.13 |
|  | 0.6 | 0.70 | 0.30 | 0.05 | 0.05 |
|  | 0.5 | 0.58 | 0.42 | 0.12 | 0.12 |
|  | 0.4 | 0.31 | 0.69 | 0.07 | 0.07 |
|  | 0.3 | 0.35 | 0.65 | 0.12 | 0.12 |

Using the procedure described in the methods section above, the proportion of labeled cells per slide and standard error was calculated for each age group. The final values used to construct Fig. 1H are included in the tables below. Standard error is abbreviated to SE, the proportion of Area X labeled cells per slide is abbreviated to PX, and the proportion of MST labeled cells per slide is abbreviated to PMST.
